# Dissecting the pathogenic effects of smoking and its hallmarks in blood DNA methylation on colorectal cancer risk

**DOI:** 10.1038/s41416-023-02397-6

**Published:** 2023-08-22

**Authors:** Xuan Zhou, Qian Xiao, Fangyuan Jiang, Jing Sun, Lijuan Wang, Lili Yu, Yajing Zhou, Jianhui Zhao, Han Zhang, Shuai Yuan, Maria Timofeeva, Athina Spiliopoulou, Ines Mesa-Eguiagaray, Susan M. Farrington, Philip J. Law, Richard S. Houlston, Kefeng Ding, Malcolm G. Dunlop, Evropi Theodoratou, Xue Li

**Affiliations:** 1 Department of Big Data in Health Science School of Public Health, and Centre of Clinical Big Data and Analytics of The Second Affiliated Hospital, Zhejiang University School of Medicine, Hangzhou, China; 2 Centre for Population Health Sciences, Usher Institute, University of Edinburgh, Edinburgh, UK; 3 Colorectal Surgery and Oncology, Key Laboratory of Cancer Prevention and Intervention, Ministry of Education, The Second Affiliated Hospital, Zhejiang University School of Medicine, Hangzhou, China; 4 Centre for Global Health, Usher Institute, University of Edinburgh, Edinburgh, UK; 5 College of Public Health, Zhengzhou University, Zhengzhou, China; 6 Unit of Cardiovascular and Nutritional Epidemiology, Institute of Environmental Medicine, Karolinska Institutet, Stockholm, Sweden; 7 Danish Institute for Advanced Study (DIAS), Epidemiology, Biostatistics and Biodemography Research Unit, Institute of Public Health, University of Southern Denmark, Odense, Denmark; 8 Cancer Research UK Edinburgh Cancer Research Centre, Institute of Genetics and Cancer, University of Edinburgh, Edinburgh, UK; 9 Division of Genetics and Epidemiology, The Institute of Cancer Research, London, UK; 10 Colon Cancer Genetics Group, Institute of Genetics and Cancer, University of Edinburgh, Edinburgh, UK

**Keywords:** Genetics research, Epidemiology

## Abstract

**Background:**

Tobacco smoking is suggested as a risk factor for colorectal cancer (CRC), but the complex relationship and the potential pathway are not fully understood.

**Methods:**

We performed two-sample Mendelian randomisation (MR) analyses with genetic instruments for smoking behaviours and related DNA methylation in blood and summary-level GWAS data of colorectal cancer to disentangle the relationship. Colocalization analyses and prospective gene-environment interaction analyses were also conducted as replication.

**Results:**

Convincing evidence was identified for the pathogenic effect of smoking initiation on CRC risk and suggestive evidence was observed for the protective effect of smoking cessation in the univariable MR analyses. Multivariable MR analysis revealed that these associations were independent of other smoking phenotypes and alcohol drinking. Genetically predicted methylation at CpG site cg17823346 [*ZMIZ1*] were identified to decrease CRC risk; while genetically predicted methylation at cg02149899 would increase CRC risk. Colocalization and gene-environment interaction analyses added further evidence to the relationship between epigenetic modification at cg17823346 [*ZMIZ1*] as well as cg02149899 and CRC risk.

**Discussion:**

Our study confirms the significant association between tobacco smoking, DNA methylation and CRC risk and yields a novel insight into the pathogenic effect of tobacco smoking on CRC risk.

## Introduction

Colorectal cancer (CRC) is the third most diagnosed cancer worldwide, and it is also the second leading cause of cancer related death [1]. Tobacco smoking had been reported to be robustly associated with CRC risk both in a binary and a dose-dependent manner in numerous observational studies. A recent meta-analysis reported that ever smokers conferred 17% additional risk of CRC in comparison to never smokers and the risk of CRC increased linearly with increasing smoking intensity, duration, and pack years of smoking [2]. However, it is hard to control for all confounders in observational studies since tobacco smoking correlates with many lifestyle and socioeconomic factors.

Mendelian randomisation (MR) is a method commonly applied in epidemiology to estimate the causal relationship between a modifiable risk factor and a health related trait or disease with genetic variants utilised as instrumental variables (IVs) [3]. Several MR studies have explored the relationship between tobacco smoking and CRC risk, but their findings and conclusions are inconsistent [4–6]. In addition, the potential mechanism by which smoking modulates the risk of CRC is not fully understood.

A number of epigenome-wide association studies (EWAS) have suggested that smoking is responsible for changes in DNA methylation across the whole epigenome, particularly at the aryl hydrocarbon receptor repressor (*AHRR*) gene locus [7–11]. Meanwhile, changes in DNA methylation are also associated with the development of CRC [12, 13]. The hypermethylation at CpG sites located in the promoter region is associated with transcriptional suppression of tumour-suppressor genes in cancer cells, especially in the case of CRC, and genome-wide hypomethylation is also one of the aberrant methylation events in CRC [13]. It is therefore hypothesised that DNA methylation might be a responsive epigenetic pathway, which bridges the genetic susceptibility of CRC with exposure to tobacco smoking.

In this study, we aimed to comprehensively disentangle the complex relationship between genetic predisposition to smoking behaviours and CRC risk and to investigate the effects of genetically predicted smoking-related methylation on CRC risk using two-sample MR analyses. We then performed genetic colocalization analyses and gene-environment interaction analyses to provide insight into how smoking may exert its carcinogenesis effect.

## Methods

### Genetic instruments for smoking behaviours

Genetic instruments for smoking behaviours were derived from the most updated GWAS conducted by the GWAS and Sequencing Consortium of Alcohol and Nicotine use (GSCAN) with a sample size of 3.4 million individuals of multi-ethnicity [14]. Smoking initiation traits included a continuous phenotype (age of initiation of regular smoking, *AgeSmk*) and a binary phenotype (smoking initiation [yes or no], *SmkInit*). In total, 703 and 27,974 SNPs were identified to be significantly associated with these two smoking initiation traits at genome-wide significance (*p* < 5 × 10^−8^) in European descendants respectively [14]. Comparing current versus former smokers, 2267 SNPs were identified to be associated with smoking cessation (*SmkCes*) at genome-wide significance in European descendants [14]. To assess the heaviness of smoking, the average number of cigarettes smoked per day (*CigDay*) was measured among both current and former smokers, and 4687 SNPs were identified at genome-wide significance in European descendants [14]. Besides, Wootton et al. conducted another GWAS of lifetime smoking behaviour (*SmoIndex*) which is a synthetic index on the basis of combined information on smoking intensity (number of cigarettes per day), smoking duration, and ever/never regular smoking status in a sample of 462,690 European individuals from UK Biobank, and 126 SNPs were identified at genome-wide significance [15]. To derive an independent set of genetic instruments for these five phenotypes, we excluded SNPs in linkage disequilibrium (LD, *r*^2^ > 0.01) and the ones with the smallest *p* values in relation to each phenotype were retained. Strand-ambiguous SNPs were excluded for quality control and 10, 327, 28, 60, and 120 SNPs were utilised as IVs, whose *F*-statistics were all above 10 (Table S1).

### Genetic instruments for smoking-related methylation

We obtained the effect estimates between smoking and DNA methylation from a meta-analysis of EWASs which included 15,907 participants from 16 cohorts in the Cohorts for Heart and Aging Research in Genetic Epidemiology Consortium [16]. Methylation was measured on DNA extracted from whole blood, CD4^+^T cells, or monocytes in each cohort using the Infinium HumanMethylation450 BeadChip containing 485,512 CpG sites, and the association between smoking and DNA methylation was adjusted by sex, age, technical covariates, and blood cell counts if applicable [16]. After quality control procedures, CpG sites that were available in less than three cohorts were removed, and the remaining 485,381 CpG sites were included in the meta-analysis [16]. Contrasting current versus never smokers, 2623 CpG sites annotated to 1405 genes were identified with significant associations to smoking behaviour at the Bonferroni threshold of *p* < 1 × 10^−7^ (≈0.05/485,381) [16] (Table S2).

For each of the 2623 CpG sites, we derived mQTLs robustly associated with its methylation level in whole blood from 32,851 European participants in the Genetics of DNA Methylation Consortium (GoDMC) [17]. Sex, age at measurement, batch variables, smoking and recorded cell counts were used to adjust for possible confounding and to reduce residual variation [17]. Genetic principal components, nongenetic DNA methylation principal components, and predicted smoking and cell counts were calculated and added to the regression model as additional confounders [17]. To comprehensively proxy the methylation level for each CpG site associated with smoking, we extracted both the significant *cis*-mQTL (*p* < 1 × 10^−8^, distance between mQTL and CpG site <1 MB) and trans-mQTL (*p* < 1 × 10^−14^, distance between mQTL and CpG site >1 MB) from the additive random effects meta-analysis and applied LD pruning (*r*^2^ > 0.01) for the selection of independent genetic instruments. Similarly, Strand-ambiguous SNPs were excluded for quality control. In total, 909 CpG sites with at least three IVs were finally included for the MR analyses.

### GWAS summary statistics

We derived GWAS summary statistics for CRC from a meta-analysis of 12 previously reported GWASs, comprising 20,049 cases and 22,661 controls of European ancestry from the following studies: CCRR1, CCFR2, COIN, CORSA, Croatia, DACHS, FIN, NSCCG-OncoArray, SCOT, UK1, VQ58, and Scottish case-control series [18]. After standard quality control procedures, a total of 16,871 cases and 26,328 controls were included in the meta-GWAS analysis [18]. To conduct stratification analyses on subsite, we also obtained GWAS summary statistics for colon and rectal cancer from a meta-GWAS of the UK Biobank and the Kaiser Permanente Genetic Epidemiology Research on Adult Health and Aging (GERA) cohorts. There were 3793 and 2091 cases for colon and rectal cancer respectively and 410,350 cancer free controls [19]. For each of the IVs selected for smoking behaviours and smoking-related methylation, the effect estimates (change in CRC, colon and rectal cancer risk per effect allele) along with standard errors, the effect and other alleles with allele frequencies were extracted from the GWAS summary statistics for CRC and colon and rectal cancer.

### Statistical analysis

To disentangle the relationship between genetically predicted smoking behaviours and CRC risk, we calculated the effect estimates in CRC risk per standard deviation (SD) increase in genetically predicted smoking behaviours using the Wald ratio and combined in a random effects meta-analysis after weighing each ratio estimate using the inverse variance weighted (IVW) approach. To avoid the violation of the second MR assumption, we applied the MR Egger method and tested the intercept in MR Egger regression to assess the overall horizontal pleiotropy [20]. We also performed a series of sensitivity analyses to investigate the robustness of the MR estimates using weighted median, simple mode, and weighted mode approaches [21]. Given possible instability in MR estimates, we applied the global test, outlier test, and distortion test using the MR pleiotropy residual sum and outlier (MR-PRESSO) method as an additional control for pleiotropy [22]. We also calculated the statistical power of the MR analyses using the method developed by Brion et al. [23]. Since *AgeSmk*, *SmkInit*, *SmkCes*, *CigDay* are significantly correlated to each other and also highly correlated with alcohol consumption [14], multivariable MR analyses were added to uncover the independent effects by mutually adjusting these smoking phenotypes and alcohol consumption. Stratification analyses were conducted for colon and rectal cancer. All MR analyses were performed using the “TwoSampleMR” R package [24]. For multiple testing correction in the univariable MR analyses, we considered *p* value < 0.01 (0.05/5) as convincing evidence and *p* value < 0.05 as suggestive evidence.

While appraising the effect of genetically predicted smoking-related methylation on CRC risk, each CpG site was regarded as the exposure and its proxy mQTLs were used as IVs. When there were at least three IVs, we calculated the effect estimates in CRC risk per standard deviation (SD) increase in genetically predicted DNA methylation of the CpG site using the IVW approach. We additionally undertook sensitivity analysis based on MR Egger and MR-PRESSO methods (at least four IVs) to assess the risk of horizontal pleiotropy [20, 22]. Similarly, stratification analyses on colon and rectal cancer were also performed. Regarding the multiple testing correction, the false discovery rate (FDR) was applied.

For those CpG sites convincingly associated with the risk of CRC (FDR < 0.05), we additionally performed colocalization analyses and prospective mQTL-smoking interaction analyses to replicate the MR findings. Colocalization analyses aimed to investigate whether the association with methylation level of CpG site and the association with CRC risk were driven by a shared causal variant using the “coloc” R package [25]. We extracted all available mQTLs of each of these CpG sites from GoDMC and integrated them with GWAS summary data for CRC. The posterior probability of five hypotheses were tested in the colocalization analyses: (1) H_0_, No association with either trait; (2) H_1_, Association with trait 1, not with trait 2; (3) H_2_, Association with trait 2, not with trait 1; (4) H_3_, Association with trait 1 and trait 2 via two SNPs in linkage disequilibrium; and (5) H_4_, Association with trait 1 and trait 2 via one shared SNP [25]. We considered both the summary posterior probability of H_4_ for the CpG site and the posterior probability of H_4_ for the single mQTL used as genetic IV at 80% or higher as evidence of colocalization.

**Fig. 1:**
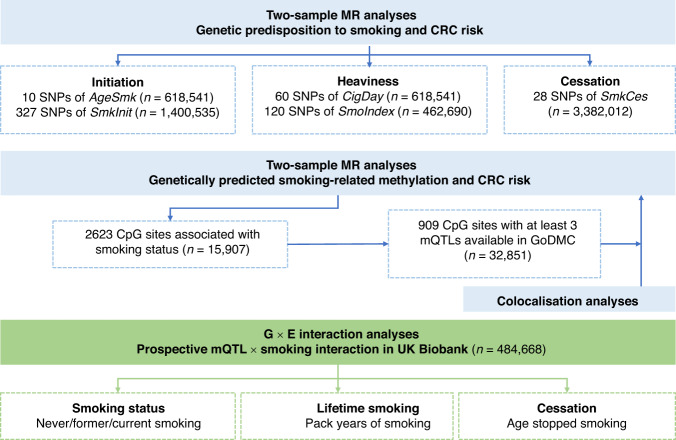
Study design. MR, Mendelian randomisation; CRC, colorectal cancer.

To conduct the prospective mQTL-smoking interaction analyses, we obtained the genotypes of mQTLs of these CpG sites along with the baseline information of three smoking phenotypes including smoking status, pack years of smoking and age stopped smoking in the UK Biobank cohort [26]. We excluded the ones with incomplete data and 6760 incident CRC cases and 477,908 non-cases were included for interaction analyses using the “CGEN” R package [27]. Age at recruitment, sex, physical activity, processed meat consumption, BMI, waist circumference, height, and the first ten genetic principal components were adjusted for potential confounding. FDR was applied for multiple testing correction. For the mQTLs that significantly interacted with all these smoking phenotypes, we further conducted stratification analyses based on their genotypes. The whole study design is presented in Fig. 1.

## Results

### Genetically predicted smoking behaviours and CRC risk

As shown in Table 1, the IVs explained 0.13–1.81% for the variances of five smoking phenotypes, with the *F*-statistics ranged from 403.52 to 14204.16. Our MR analyses identified convincing association between genetically predicted *SmkInit* and the risk of CRC. The OR and 95% CI for one-SD increase in genetically predicted *SmkInit* was 1.47 (1.24, 1.75) based on the IVW approach. This association was replicated using the weighted median and MR-PRESSO methods. Additionally, suggestive protective association (*p* < 0.05) were discovered between genetically predicted *SmkCes* and the risk of CRC, and the OR was 0.65 (95% CI: 0.43, 0.99). There was no evidence of association for genetically predicted *AgeSmk* and *CigDay*, which might be due to limited statistical power (0.20 and 0.62). Nevertheless, when using the combined *SmoIndex*, we found a suggestive effect between lifetime smoking and CRC risk at the OR of 1.30 (95% CI: 1.04, 1.62). Substantial and moderate heterogeneity was reported between the IVs of *SmkInit*, *CigDay* and *SmoIndex*, but no apparent horizontal pleiotropy or outlier was discovered using the MR Egger and MR-PRESSO approaches. In the multivariable MR analysis that mutually adjusted tobacco smoking and alcohol drinking, Table 2 displayed the independent effects of genetically predicted *SmkInit* and *SmkCes* on CRC risk. One-SD increase in genetically predicted *SmkInit* was independently associated with a higher risk of CRC with the OR of 1.51 (95% CI: 1.13, 2.03), while genetically *SmkCes* showed an independent protective effect with the OR of 0.68 (95% CI: 0.47, 0.99). These associations not only reaffirmed the pathogenic effect of tobacco use on CRC risk but also indicated the benefit of quitting smoking. When stratified on colon and rectal cancer, there was no evidence of associations for genetically predicted smoking behaviours in neither the univariable nor multivariable MR analyses, which might be due to the very limited statistical power (Tables S3 and S4).

**Table 1:** Two-sample Mendelian randomisation estimates of smoking behaviours on CRC risk.

Exposure	No. of IVs	Method	OR (95% CI)	*p*_effect_	*p*_heterogeneity_	*p*_pleiotropy_	*R*^2^	*F*-statistics	Power
*AgeSmk*	10	IVW	1.34 (0.75, 2.39)	0.330	0.616	–	0.13%	403.52	0.20
		MR Egger	0.94 (0.07, 12.48)	0.966	0.522	0.794			
		Weighted median	1.22 (0.58, 2.54)	0.604	–	–			
		Simple mode	1.15 (0.31, 4.29)	0.842	–	–			
		Weighted mode	1.14 (0.34, 3.84)	0.843	–	–			
		MR-PRESSO	1.34 (0.74, 2.41)	0.304	–	–			
*SmkInit*	327	IVW	1.47 (1.24, 1.75)	1.52 × 10^−5^	5.49 × 10^−4^	–	1.81%	14,204.16	1.00
		MR Egger	1.89 (0.87, 4.10)	0.109	5.12 × 10^−4^	0.519			
		Weighted median	1.45 (1.15, 1.83)	0.002	–	–			
		Simple mode	1.72 (0.76, 3.90)	0.194	–	–			
		Weighted mode	1.46 (0.69, 3.11)	0.327	–	–			
		MR-PRESSO	1.47 (1.23, 1.76)	2.03 × 10^−5^	–	–			
*SmkCes*	28	IVW	0.65 (0.43, 0.99)	0.044	0.067	–	0.40%	1504.37	0.76
		MR Egger	0.31 (0.11, 0.90)	0.041	0.096	0.156			
		Weighted median	0.77 (0.45, 1.33)	0.346	–	–			
		Simple mode	0.76 (0.25, 2.33)	0.638	–	–			
		Weighted mode	0.80 (0.32, 2.05)	0.654	–	–			
		MR-PRESSO	0.65 (0.42, 1.01)	0.054	–	–			
*CigDay*	60	IVW	1.20 (0.97, 1.48)	0.091	0.031	–	1.44%	4584.08	0.62
		MR Egger	1.09 (0.74, 1.61)	0.670	0.028	0.567			
		Weighted median	0.97 (0.72, 1.31)	0.838	–	–			
		Simple mode	1.30 (0.59, 2.86)	0.523	–	–			
		Weighted mode	1.09 (0.75, 1.60)	0.639	–	–			
		MR-PRESSO	1.20 (0.97, 1.49)	0.096	–	–			
*SmoIndex*	120	IVW	1.30 (1.04, 1.62)	0.023	0.033	–	1.07%	4939.85	0.81
		MR Egger	1.30 (0.53, 3.24)	0.569	0.029	0.991			
		Weighted median	1.23 (0.91, 1.66)	0.179	–	–			
		Simple mode	1.28 (0.54, 3.00)	0.577	–	–			
		Weighted mode	1.20 (0.66, 2.18)	0.561	–	–			
		MR-PRESSO	1.30 (1.03, 1.63)	0.025	–	–

**Table 2:** Multivariable Mendelian randomisation estimates of smoking behaviours on CRC risk.

Exposure	No. of IVs	OR (95% CI)	*p* value
*AgeSmk*	5	1.01 (0.63, 1.61)	0.980
*SmkInit*	305	1.51 (1.13, 2.03)	0.005
*SmkCes*	16	0.68 (0.47, 0.99)	0.042
*CigDay*	44	1.11 (0.89, 1.39)	0.355
*DrnkWk*	85	1.29 (0.97, 1.71)	0.085

OR (95% CI), independent causal effects on CRC risk when mutually adjusted tobacco smoking and alcohol drinking.

### Genetically predicted smoking-related methylation and CRC risk

Tables 3 and S5–S7 present the MR estimates of genetically predicted methylation at 909 CpG sites with at least three IVs and the risk of CRC. Based on the IVW approach, we identified that methylation at 68 smoking-related CpG sites were nominally associated with the risk of CRC (*p* < 0.05), and two of them survived multiple testing correction (FDR < 0.05). For one-SD increase in genetically predicted methylation level at CpG site cg02149899, the CRC risk would correspondingly increase with the MR estimates of 1.14 (95% CI: 1.07, 1.22). On the contrary, genetically predicted methylation at CpG site cg17823346 [*ZMIZ1*] was linked to a decreased risk of CRC with the MR estimate of 0.88 (95% CI: 0.84, 0.93). Stratification on subsite discovered 42 and 49 CpG sites nominally associated with the risk of colon and rectal cancer, and five of them were overlapping (Table S8). None of these associations passed multiple testing correction using FDR.

**Table 3:** Two-sample Mendelian randomisation estimates of smoking-related methylation on CRC risk.

CpG site	Locus	Gene	Smoking-CpG estimates	No. of IVs	Method	OR (95% CI)	*p* value	FDR
cg17823346	10:80848143-80848143	*ZMIZ1*	−0.013	5	IVW	0.88 (0.84, 0.93)	8.97 × 10^−6^	0.008
cg02149899	11:75993521-75993521	*–*	−0.007	5	IVW	1.14 (1.07, 1.22)	8.92 × 10^−5^	0.041

Smoking-CpG estimates, the effect estimates of the associations between smoking status and CpG site methylation; OR (95% CI), CRC risk for per SD increase in genetically predicted CpG site methylation.

**Fig. 2:**
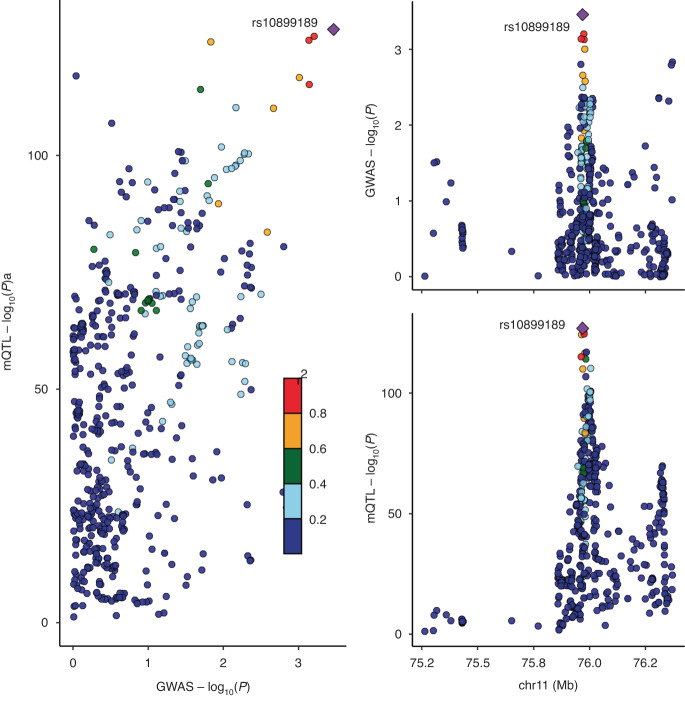
Regional plot of colocalization evidence of CpG site methylation and CRC susceptibility. Strong evidence supports rs10899189 as a shared causal variant underlying methylation at cg02149899 and CRC susceptibility (posterior probability = 97.8%).

For the replication of the two CpG sites convincing associated with CRC risk, we observed strong colocalization evidence for cg02149899 and CRC risk. Figure 2 suggested that methylation at cg02149899 and CRC GWAS signals had 97.8% posterior probability of sharing a causal variant (rs10899189). However, methylation at cg17823346 and CRC susceptibility were observed to be driven by distinct SNPs in linkage disequilibrium (PPH_3_ > 80%). For the interaction analyses, the baseline characteristics of CRC incident cases and non-cases in UK Biobank are summarised in Table S9. Table S10 displays the prospective mQTL-smoking interaction effect estimates on CRC risk. Four mQTLs including rs12263636 of cg17823346, and rs616263, rs10899189, and rs2618091 of cg02149899 had evidence for interaction across all three smoking phenotypes. We further performed stratification analyses based on the genotypes of these four mQTLs (Table S11). Despite carrying no risk allele of CRC, current smokers with rs616263 CC genotypes conferred 43% higher risk of CRC compared to non-smokers, with a relative risk (RR) of 1.43 (95% CI: 1.19, 1.72); and current smokers with rs10899189 TT genotypes was associated with 13% higher risk of quitting smoking every 10 years later.

## Discussion

In our study, we comprehensively examined the relationship between tobacco smoking, smoking-related DNA methylation and CRC risk via MR analyses. We additionally performed genetic colocalization analyses and gene-environment interaction analyses to unveil how tobacco smoking modulates the risk of CRC in the context of epigenetic modifications.

We detected convincing evidence in support of the pathogenic effect of smoking initiation on CRC risk and suggestive evidence for the protective effect of smoking cessation in the univariable MR analyses. Moreover, these associations were proved to be independent of other smoking phenotypes and alcohol drinking in the multivariable MR analysis. Nevertheless, these significant findings in binary exposures should be interpreted with caution, given that binary exposures in MR analyses might violate the core assumptions of instrumental variables and bias the true effect estimates [28, 29]. To complement the drawback, we also included two continuous exposures (*CigDay* and *SmoIndex*) in the univariable MR analyses. Weak evidence supporting the association between *CigDay* and CRC risk is probably due to the limited statistical power. However, we identified that lifetime smoking was suggestively associated with CRC risk using the combined *SmoIndex*. This finding is in the consistent direction, but a larger estimate and wider confidence interval compared with the MR study conducted by Dimou et al. [5], where they used larger CRC GWAS summary statistics. No evidence supported the effects of smoking behaviours on colon or rectal cancer, which might be due to the small number of cases and limited statistical power. Additionally, the GWAS on tobacco smoking and the GWAS on colon and rectal cancer had overlapping participants from the UK Biobank. If the GWAS on tobacco smoking included both the individuals with colon and rectal cancer and cancer free controls from the UK Biobank, the null MR estimates would be biased due to sample overlap. This bias is a linear function of the proportion of overlap between these two samples [30].

When exploring the effects of genetically predicted smoking-related methylation on CRC risk, we discovered two CpG sites, cg17823346 [*ZMIZ1*] and cg02149899, whose methylation were found to modulate CRC risk through epigenetic modification. Genetically predicted methylation at cg17823346 [*ZMIZ1*] was linked to a decreased risk of CRC while genetically predicted methylation at cg02149899 was correlated with an elevated risk of CRC. In the replication analyses with colocalization and gene-environment interaction approaches, strong colocalization evidence was observed for methylation at cg02149899 and CRC susceptibility, and significant mQTL-smoking interaction was identified for both of these two CpG sites.

Smoking had been reported in association to the incidence of CRC with fewer T cells and tumour associated macrophages infiltration [31–33], suggesting the possible mechanism that smoking modifies the risk of CRC via the suppression of anti-tumour immunity. In the meantime, T cells and tumour associated macrophages in the colorectal tumour microenvironment come from bone marrow and blood, smoking-related DNA methylation in blood may be possibly linked to these phenomena of immune suppression and evasion. CpG site cg17823346 is mapped to gene *ZMIZ1*, which encodes a transcriptional co-activator in the protein inhibitor of activated STAT (PIAS)-like family [34]. Protein ZMIZ1 can directly interact with protein Notch1 through a tetratricopeptide repeat domain without affecting intestinal homoeostasis or myeloid suppression, and selectively regulates the expression of Notch1 target genes, especially *Myc* [35]. The Zmiz1-Notch1 protein-protein interaction is also of great importance for the normal proliferation of T cell precursors, and disruption of this homoeostasis leads to the development of leukaemia [36]. Moreover, rs704071 located on the antisense RNA of *ZMIZ1* (*ZMIZ1-AS1*) had been identified in relation to the genetic susceptibility of CRC in East Asians [37], and it should also be noted that the risk allele G of this SNP is associated with a lower risk of early-onset CRC but a higher risk of late-onset CRC [38]. Evidence from our methylation MR and gene-environment interaction analyses provided a novel insight into the role of gene *ZMIZ1* in the development of CRC from the perspective of DNA methylation. The CpG site cg02149899 had no mapped genes, but our study provided strong evidence for its pathogenic effects on CRC utilising the mQTLs and future wet-lab functional experiment is of great importance to validate our findings and to further interpret its role in the development of CRC.

The strengths of our study include the systematic evaluation of the complex relationship between smoking behaviours, blood DNA methylation, and CRC risk within the framework of two-sample MR analyses. Furthermore, we applied genetic colocalization and gene-environment interaction analyses to explore the possible mechanisms by which tobacco smoking exerts its carcinogenesis in the context of DNA methylation. Nevertheless, there are several potential limitations in our study. The CpG sites associated with smoking status were derived from a cross-sectional EWAS, which limited the possibility to investigate the time course of tobacco smoking on DNA methylation [16]. In addition, our study utilised CpG sites and mQTLs in DNA samples from blood [16, 17]. Despite the advantages of ease to access and non-invasive sample collection, DNA methylation signature differs across tissues, and the microenvironment in blood is quite different from that in colonic epithelia. Therefore, it would be worth conducting further research using data from colon tissues. Without access to the GWAS summary statistics for DNA methylation, we were not able to estimate how much DNA methylation mediates the pathogenic effect of smoking on CRC risk using the multivariable MR analyses.

In conclusion, our study provided convincing evidence to support the pathogenic effect of smoking initiation on CRC risk and suggestive evidence for the protective effect of smoking cessation. These associations were independent of other smoking phenotypes and alcohol consumption. Using mQTLs as proxies for CpG site methylation, we found that the pathogenic effect of tobacco smoking on CRC risk could be partly attributed to epigenetic modification at two CpG sites and mapped genes.

### Supplementary information

Supplementary tables

## Data Availability

The GWAS for tobacco smoking and alcohol drinking can be obtained through the GSCAN data portal (https://conservancy.umn.edu/handle/11299/241912) and the dataset on the website of the University of Bristol (https://data.bris.ac.uk/data/dataset/10i96zb8gm0j81yz0q6ztei23d). The mQTLs were derived from the GoDMC consortium (http://mqtldb.godmc.org.uk/). CRC summary statistics were generated based on 12 previous GWASs (https://onlinelibrary.wiley.com/doi/10.1002/ijc.33191). The UK Biobank data were derived under Application Number 66354. All data generated in the current study can be obtained from the Supplementary files.
